# Clinical practice guidelines for the treatment of tardive dyskinesia in Europe: A descriptive review

**DOI:** 10.1192/j.eurpsy.2025.10047

**Published:** 2025-07-17

**Authors:** Mark J. Edwards, Pierre Michel Llorca, Maurice T. Driessen, Krzysztof Duma, Nayla Chaijale, Liza Sopina, Sameer Kotak, Andrea Fagiolini, David Taylor, Christoph U. Correll

**Affiliations:** 1 Department of Basic and Clinical Neuroscience, Institute of Psychiatry, Psychology and Neuroscience, King’s College London, London, UK; 2 Department of Medical and Research Activities, Fondation FondaMental, Créteil, France; 3 Department of Psychiatry, Clermont-Ferrand University Hospital Center, Clermont-Ferrand, France; 4 Department of Psychiatry, University of Clermont Auvergne, Clermont-Ferrand, France; 5 Global Health Economics and Outcomes Research, Teva Pharmaceuticals EU, Amsterdam, Netherlands; 6 Movement Disorders, Teva Pharmaceuticals EU, Amsterdam, Netherlands; 7 Global Medical Affairs, Teva Branded Pharmaceutical Products R&D LLC, West Chester, PA, USA; 8 Independent Consultant, Odense, Denmark; 9 Management, Yorker Health, Glen Rock, NJ, USA; 10 Department of Molecular and Developmental Medicine, University of Siena, Siena, Italy; 11 Pharmaceutical Science, King’s College London, London, UK; 12 Department of Psychiatry, The Zucker Hillside Hospital, Northwell Health, Glen Oaks, NY, USA; 13 Department of Psychiatry and Molecular Medicine, Donald and Barbara Zucker School of Medicine at Hofstra/Northwell, Hempstead, NY, USA; 14 Department of Psychiatric Neuroscience, Feinstein Institutes for Medical Research, Institute of Behavioral Science, Manhasset, NY, USA; 15 Department of Child and Adolescent Psychiatry, Psychiatry, Charité – Universitätsmedizin Berlin, Berlin, Germany; 16 Department of Psychiatry, German Center for Mental Health (DZPG), Berlin, Germany

**Keywords:** antipsychotic medication, clinical practice guidelines, tardive dyskinesia, treatment recommendations, VMAT2 inhibitor

## Abstract

**Background:**

While evidence-based clinical practice guidelines promote high-quality care, their absence may create unwarranted variation in disease management, leading to suboptimal outcomes. This study aimed to identify existing clinical practice guidelines for tardive dyskinesia (TD) in France, Germany, Italy, Spain, and the United Kingdom (EU4+UK), assessing the evidence for recommended TD treatments.

**Methods:**

MEDLINE, PubMed, and other sources (e.g., government/public agencies, associations, patient/research organizations) were searched to identify clinical practice guidelines for TD published between January 2000 and February 2025 in EU4+UK. Mentions of TD treatments in identified documents were classified as “recommendations” or “descriptions.” Recommendations were ranked according to the Scottish Intercollegiate Guidelines Network grading system or received a “no-rank” label. Subanalyses on tetrabenazine and tiapride, were performed.

**Results:**

Of the 31 documents identified, only two were TD-specific, with the remainder primarily developed for schizophrenia, major depressive disorder, and bipolar disorder. Data extraction led to 112 mentions of TD treatments (40 recommendations, 72 descriptions). Most recommendations focused on antipsychotic regimen modification (75%) and had no rank (88%). Only five recommendations (no rank) proposed a pharmaceutical (add-on) treatment: three for tetrabenazine and one each for amantadine and buspirone. Neither of the TD-specific guidelines contained TD treatment recommendations.

**Conclusions:**

No specific clinical practice guidelines for TD in EU4 + UK were found, although TD management was mentioned in guidelines for other disorders. Most recommendations were not supported by high-quality evidence. To improve quality of care for patients with TD in Europe, updated treatment recommendations are needed based on high-quality studies.

## Introduction

Tardive dyskinesia (TD) is a serious drug-induced movement disorder characterized by abnormal, involuntary hyperkinetic movements; these movements typically involve the tongue, lower face, and jaw, but may also affect the extremities and/or pharyngeal, diaphragmatic, and trunk muscles [1]. TD is caused by long-term exposure to dopamine receptor antagonists, primarily antipsychotic medications (APs), and is therefore common among patients who are treated with APs for schizophrenia, bipolar disorder, and major depressive disorder [1]. As APs are often used off-label in clinical practice, patients with other conditions may also develop TD [2–6].

TD is estimated to affect up to 25% of patients treated with APs globally, with higher rates observed among older individuals and those with prolonged AP exposure [7]. The prevalence of TD among patients treated with first-generation APs is estimated to be higher than among those treated with second-generation APs [7, 8]. Despite the more frequent use of second-generation APs, the prevalence of TD has not decreased [9]. TD is often irreversible, imposes substantial functional impairment, and diminishes quality of life [10–13]. Real-world studies have shown an association between TD and increased healthcare resource utilization and healthcare costs [14, 15].

Guidelines for the appropriate management of TD are therefore important to reduce disease burden. Clinical practice guidelines are defined by the Institute of Medicine (IoM) as “statements that include recommendations, intended to optimize patient care, that are informed by a systematic review of evidence and an assessment of the benefits and harms of alternative care options” [16, 17]; these statements can either be “recommendations” or “descriptions” for disease management strategies and pharmacological treatment [18]. The absence of international or local clinical practice guidelines can create unwarranted variation in disease management and may lead to suboptimal outcomes for patients [19].

TD-specific clinical practice guidelines are available in some countries. For example, in the United States (US), the American Academy of Neurology published guidelines [20], which were followed by recommendations in Japan and Canada [21, 22]. In addition, the American Psychiatric Association (APA) has published TD-specific clinical practice guidelines and management strategies in the *APA Practice Guideline for the Treatment of Patients With Schizophrenia* [23]. However, clinical practice guidelines for TD management and associated treatment recommendations in Europe are limited. The current targeted literature review aimed to identify and compare existing clinical practice guidelines addressing TD in France, Germany, Italy, Spain, and the United Kingdom (EU4 + UK), and to assess the evidence for their recommended TD treatment options.

## Methods

### Search strategy

Electronic databases, such as MEDLINE, PubMed, and other sources (e.g., government/public agencies, associations, patient/research organizations), without language restrictions, were searched for clinical practice guidelines for TD published between January 1, 2000, and February 21, 2025, in EU4 + UK; a list of organizations by country is provided in Supplementary Table 1. MEDLINE and PubMed keyword searches on abstracts and titles were conducted for updates on TD clinical practice guidelines for each country (Supplementary Table 2) using country terms and TD terms composed of Medical Subject Headings terms.

A grey literature search using the same terminology was also conducted. The search commenced after establishing a list of potential sources of guidelines by evaluating the types of organizations, bodies, and entities that have created clinical guidelines in other therapeutic areas. Examples include government or public agencies, reimbursement agencies, medical associations, neurological associations, psychiatric associations, patient organizations, and research organizations, among others. Next, the types of organizations existing within each of the five countries were identified via online searches of local Google sites using key terms, as well as searches for relevant associations or organizations in Europe, in addition to academic conferences and journals stemming from such organizations. Once a comprehensive list of potential source organizations was created for each country, each one was searched for mention of TD. This search was completed by utilizing the search feature of each organization as well as a Google search within that webpage. An additional gray literature search was performed by searching for TD combined with each country, without restriction to any specific organization.

### Inclusion criteria

To ensure that all relevant publications were captured, the IoM definition of “clinical practice guideline” was broadened to include systematic reviews and consensus statements (e.g., Delphi panel studies). Treatment-level data and studies (e.g., randomized clinical trials) were excluded. Analyses were restricted to the year of the earliest guideline document reviewed for that country.

Clinical practice guidelines were included in this review if they met the following criteria: public availability (i.e., a resource that could be obtained by most clinicians or researchers); contained guidance on the prevention, assessment, diagnosis, treatment, or management of TD; authored by a professional body, institution, organization, or entity that is regionally or nationally recognized as a subject matter authority by the healthcare community; and supported by studies (industry-sponsored documents were excluded under this definition).

### Data extraction

Seventeen documents in languages other than English were machine-translated into English and subsequently reviewed by a native speaking author. The following data, if reported, were extracted: title of guideline; year of publication; governing body; composition of the panel; summary of the content; information on TD prevention, assessment, diagnosis, or management (e.g., AP cessation, switch, or dose reduction or increase; intervention, including any pharmacological or non-pharmacological treatment), and literature/evidence cited, including levels of recommendation and supporting evidence.

All TD-related mentions concerning the four main topics (prevention, assessment, diagnosis, or management) and details on management strategies were further classified as either a “recommendation” or a “description.” Recommendations contained information that directly informs clinical practice and were defined by specific use of “recommendation” or “instruction” terms, per the definition of “clinical practice guideline” previously provided. Descriptions contained any reference or mention of TD treatment without an accompanying recommendation.

### Grading of TD recommendations and level of evidence assessment

Extracted data were assigned a grade based on the Scottish Intercollegiate Guidelines Network (SIGN) classification, a rigorous system designed to assess the strength of recommendations based on the quality of supporting evidence [24]. The quality of evidence used to support treatment recommendations was assigned numeric levels ranging from 1++ to 4 based on study design, consistency, and applicability of evidence (Supplementary Table 3). Level 1++ signifies high-quality evidence, such as comprehensive meta-analyses, systematic reviews of randomized clinical trials, and randomized clinical trials with a very low risk of bias. Level 4 represents weak evidence, such as expert opinion ( Supplementary Table 3).

Subsequently, recommendations were rated A, B, C, or D, indicating the extent to which healthcare professionals should consider adhering to the recommendation [24], with grade A signifying a strong recommendation based on high-quality evidence and grade D reflecting a weak recommendation supported by low-quality evidence or expert opinion ( Supplementary Table 4). For treatment recommendations where a grade was provided by guideline authors, those grades were used. Most graded recommendations followed the SIGN grading system. In instances where the SIGN grading system was not utilized, the grades provided were assigned the closest SIGN grading, following the rules described in papers authored by the GRADE working group [25]. For treatment recommendations that did not have a grade, an additional “no-rank” label was added, which is not included in the SIGN grading system.

### Statistical analysis

This systematic literature review was a descriptive study, with no statistical analyses conducted. Information was compared descriptively across countries, treatments, and treatment categories.

### Subgroup analysis of literature informing guideline recommendations on the use of tetrabenazine and tiapride

In some EU4 + UK countries, both tetrabenazine and tiapride are indicated for the treatment of TD. Therefore, there was interest in evaluating the quality of the evidence supporting recommendations of these pharmacological treatments. As such, any literature (e.g., clinical studies, case series, systematic reviews) that informed guideline recommendations for these agents was also analyzed.

## Results

### Clinical practice guideline identification

In total, 31 of the documents identified met the definition of a “clinical practice guideline,” including one non-industry-sponsored continuing medical education series certified by the German Medical Association (Table 1 and Supplementary Table 1). Of these 31 documents, 22 were identified from searching 56 health-related organizations, eight were added after review by country experts and authors, and one was identified through MEDLINE and PubMed searches (search strategy described in Supplementary Table 2). Several documents identified through peer-reviewed literature searches and more generic searches were excluded because they were not specific to at least one EU4 + UK country.

**Table 1.** Clinical practice guidelines by country, indications, and years published

Country	Organization (*n*)	Guideline documents					Range of years guidelines were published
		TD	Schizophrenia	Major depressive disorder	Bipolar disorder	Total	
France	8	0	2	0	0	2	2017–2022
Germany	11	1	3	0	1	5	2018–2021
Italy	9	0	2	0	0	2	2002–2007
Spain	11	1	5	0	1	7	2009–2021
UK	17	0	12	0	3	15	2013–2025
Total	56	2	24	0	5	31	2002–2025

Abbreviations: TD, tardive dyskinesia; UK, United Kingdom.

### Clinical practice guideline characteristics

Only two documents were TD-specific (one from Germany and the other from Spain), although neither contained recommendations (Table 1). The other documents (*n* = 29) reporting on the prevention, assessment, diagnosis, or treatment of TD were guidelines primarily developed for the management of patients with schizophrenia, major depressive disorder, or bipolar disorder.

The country with the most identified documents was the UK (*n* = 15), followed by Spain (*n* = 7), Germany (*n* = 5), and France and Italy (both *n* = 2). The clinical practice guidelines for Germany, the UK, France, and Spain were relatively up to date, with the oldest guidelines released in 2021; only the guidelines for Italy had not been updated since 2007 (Table 1).

### Categorization of TD treatment and management strategies

**Figure 1.**
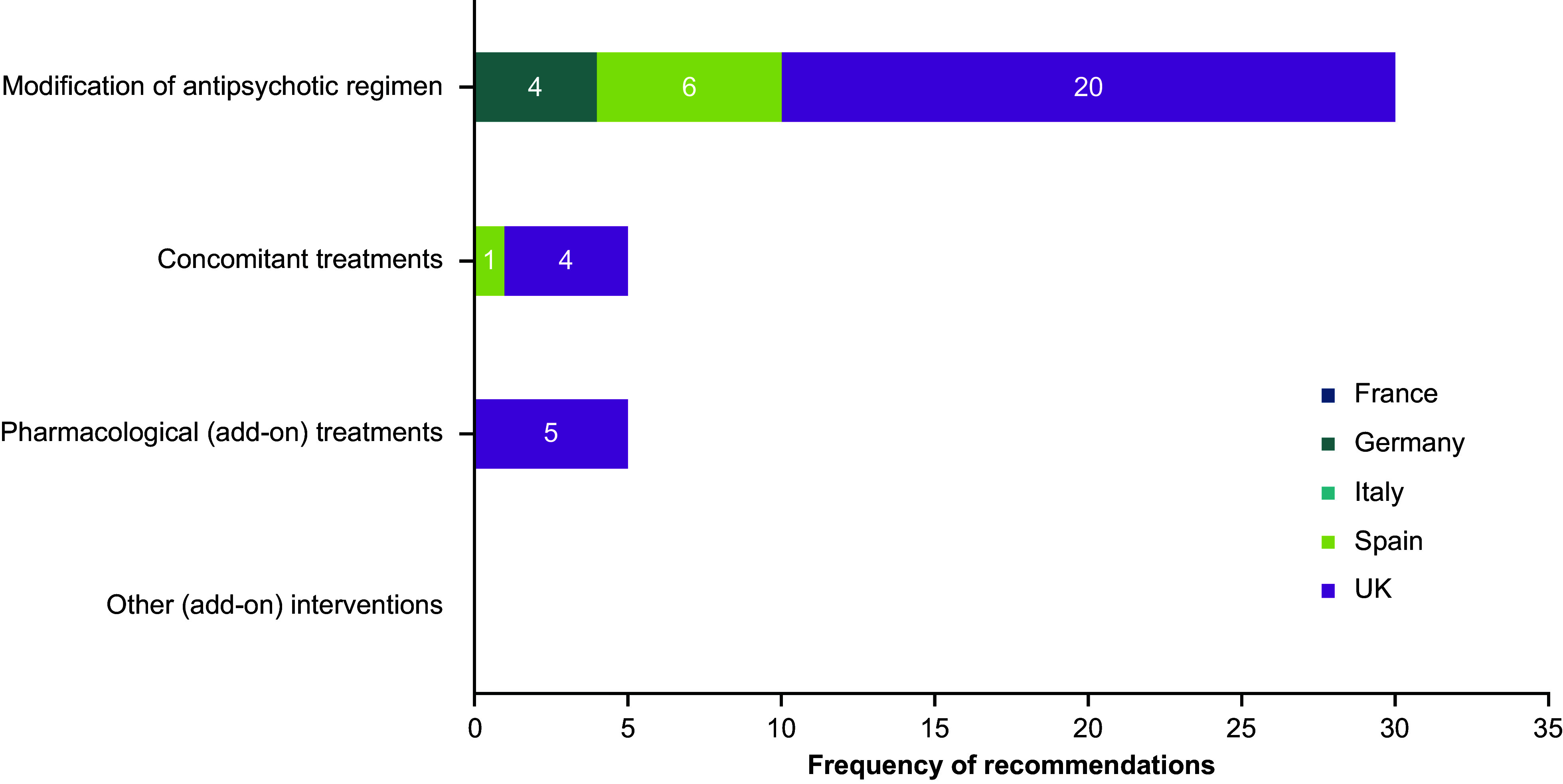
TD treatment recommendations by country, clinical practice guidelines, and treatment category. Abbreviations: TD, tardive dyskinesia; UK, United Kingdom.

**Figure 2.**
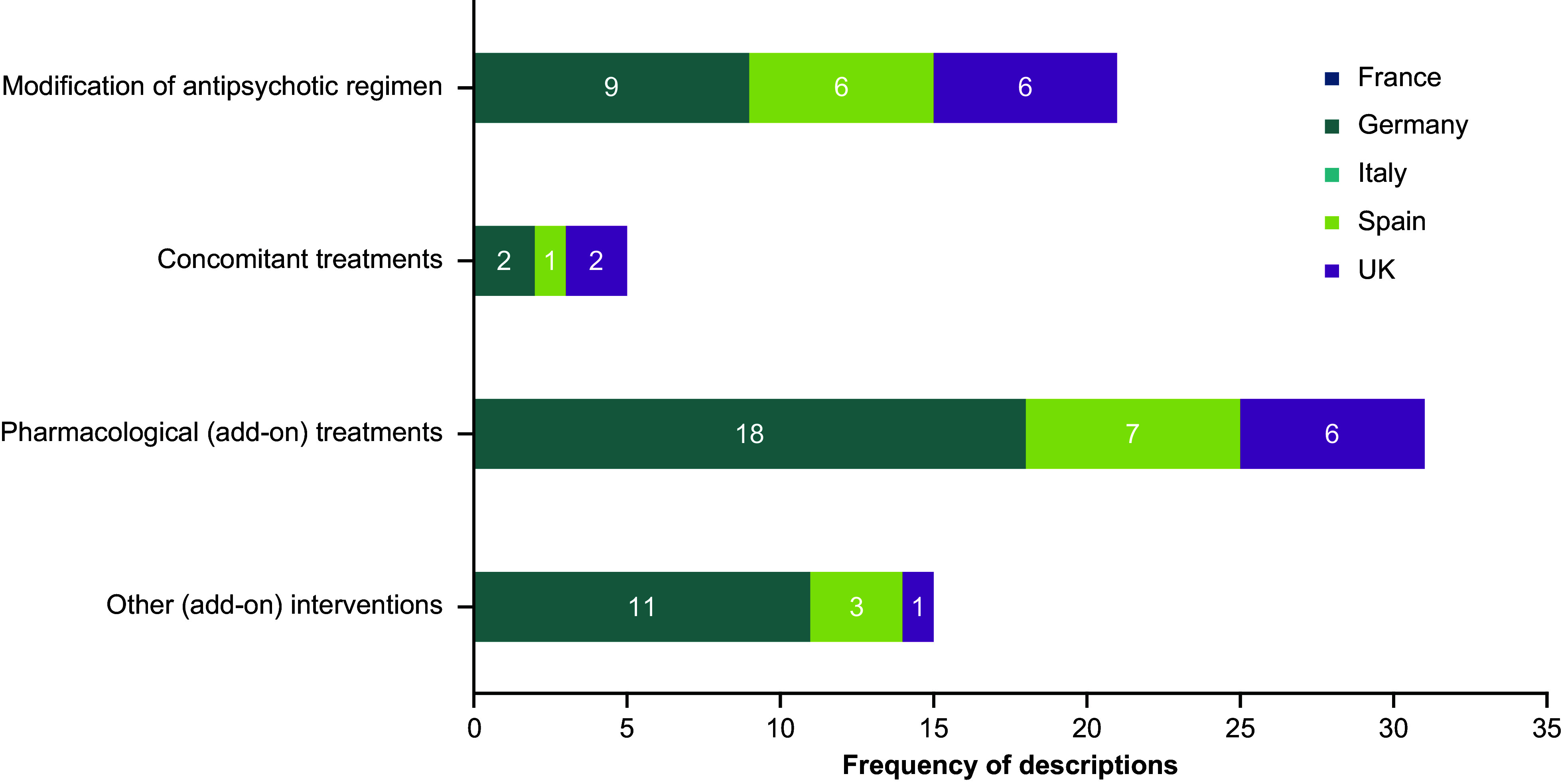
TD treatment descriptions by country, clinical practice guidelines, and treatment category. Abbreviations: TD, tardive dyskinesia; UK, United Kingdom.

A total of 112 TD-related mentions were identified during the data extraction process, with 40 classified as “recommendations” and 72 as “descriptions.” Of the 40 recommendations, most were from the UK (*n* = 29), followed by Spain (*n* = 7) and Germany (*n* = 4); no recommendations were identified in guidelines from France or Italy (Figure 1). Of the 72 descriptions, Germany had the most (*n* = 40), followed by Spain (*n* = 17) and the UK (*n* = 15); no descriptions were identified in guidelines from either France or Italy (Figure 2).

The 112 TD treatment and management strategies were grouped into four high-level categories ( Supplementary Table 5): modification of AP regimen (dose change, treatment switch, or cessation); pharmacological (add-on) treatments; non-pharmacological or alternative treatments; and changing concomitant treatments other than APs. Table 2 shows the breakdown into the four categories for both recommendations and descriptions. No recommendation or description appeared more than five times, with many appearing only once.

**Table 2.** Summary of TD treatment descriptions and recommendations by category

Category	Type of TD management	Description	Unranked recommendation	Recommendations with grades^a^
Modification of AP regimen	Clozapine	5	5	0
	Reduce AP dose	3	4	1 (D)
	Unspecified change/alternative second-generation AP	1	3	3 (A, B, D)
	Quetiapine	3	3	0
	Olanzapine	4	3	0
	Discontinue AP	1	4	1 (D)
	Aripiprazole	0	2	0
	Increase AP dose	2	0	0
	Change to injectable	0	1	0
	Risperidone	1	0	0
	Unspecified dose change	1	0	0
Pharmacological add-on treatments	Tetrabenazine	4	3	0
	Amantadine	2	1	0
	Calcium antagonists	3	0	0
	GABAergic agonists/benzodiazepines	3	0	0
	Valbenazine	3	0	0
	Buspirone	1	1	0
	Catecholaminergic drugs	2	0	0
	Deutetrabenazine	2	0	0
	Donepezil/cholinergics	2	0	0
	Tiapride	2	0	0
	VMAT2 inhibitor (non-specific)	2	0	0
	Cholinergic agonists	1	0	0
	Cholinesterase inhibitors	1	0	0
	L-Dopa	1	0	0
	Non-benzodiazepine GABAergic agonists	1	0	0
	Propranolol	1	0	0
Changing concomitant treatments	Stop/decrease anticholinergic	4	5	0
	Change/stop antimuscarinic	1	0	0
Other interventions	Deep brain stimulation	3	0	0
	Vitamin E	3	0	0
	Vitamin B	2	0	0
	Gingko biloba	2	0	0
	Botulinum/Botox	1	0	0
	Branched-chain amino acids	1	0	0
	Clonidine	1	0	0
	Electroconvulsive therapy	1	0	0
	Hypnosis/relaxation	1	0	0

Abbreviations: AP, antipsychotic; TD, tardive dyskinesia; VMAT2, vesicular monoamine transporter 2.

The strength of each description and recommendation is color coded; no shading represents no description or recommendation, light shading represents one description or recommendation, and medium shading represents two to five descriptions or recommendations. No description or recommendation was mentioned more than five times.

a The strength/grading of recommendations for treatment, as provided by the source, ranked from A (strongest evidence) to D (weakest evidence).

The “modification of AP regimen” category was the most common, representing 46% (51/112) of the TD-related mentions. Of these 51 mentions, 26 appeared in clinical practice guidelines from the UK, followed by 13 from Germany and 12 from Spain. Thirty mentions of the AP regimen modification were recommendations, while 21 were descriptions. Indeed, “modification of AP regimen” constituted the majority ([75% [30/40]) of all recommendations identified, with most recommending a change from a first-generation to second-generation AP. Among second-generation APs, clozapine and olanzapine were the most frequently described. Five of the “modification of AP regimen” recommendations had rankings assigned (Table 2). Three recommended changing to a second-generation AP, and were ranked “A,” “B,” and “D,” respectively; the other two were graded “D,” one of which recommended AP discontinuation and the other dose reduction.

**Figure 3.**
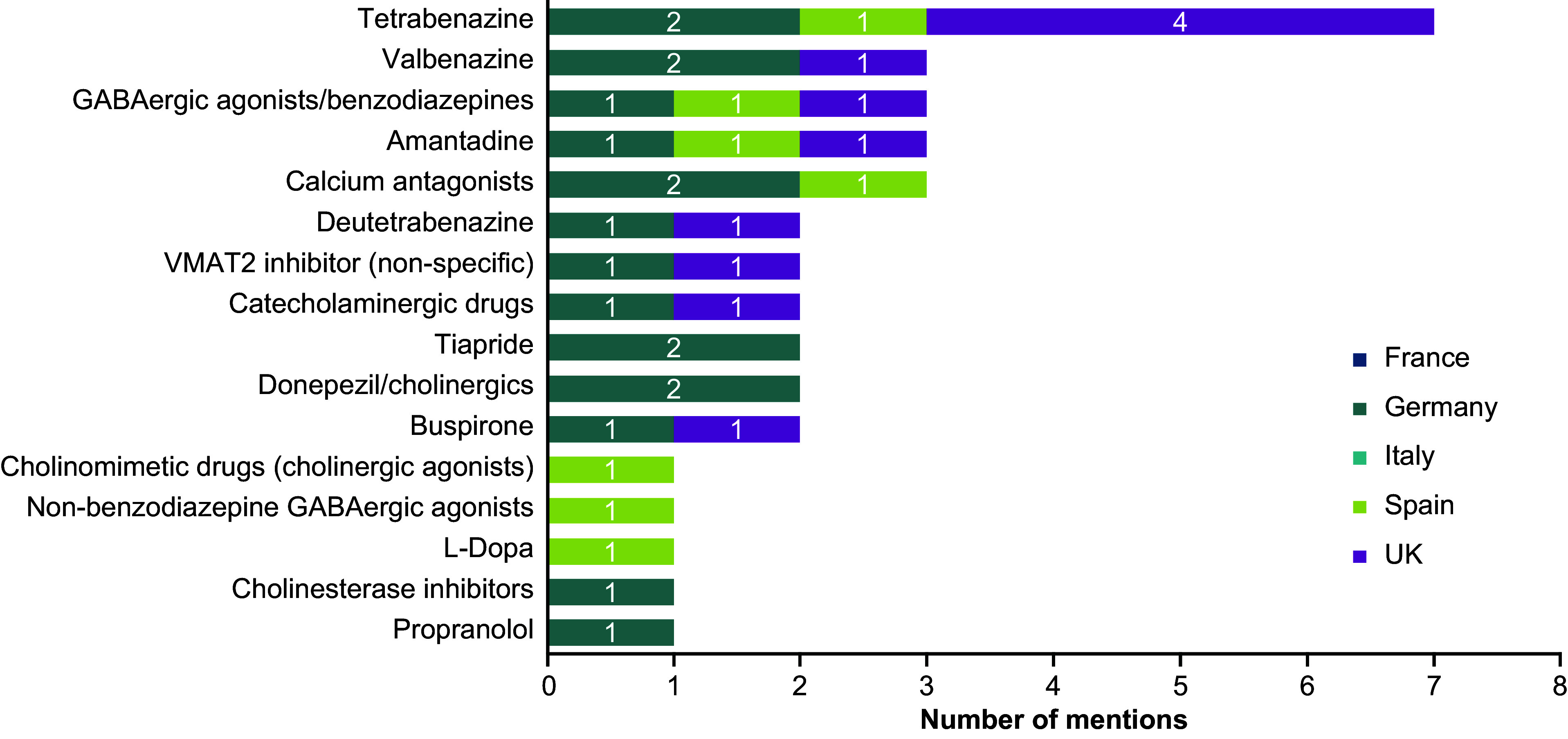
Identified recommendations and descriptions for pharmacological (add-on) treatments specific to TD by country. Abbreviations: TD, tardive dyskinesia; UK, United Kingdom; VMAT2, vesicular monoamine transporter 2.

Of the 112 TD-related mentions, 36 were in the “pharmacological (add-on) treatments” category. Of these 36 mentions, 18 were extracted from documents from Germany, 11 from the UK, and seven from Spain. The most common mentions of pharmacological (add-on) treatments referred to the vesicular monoamine transporter 2 (VMAT2) inhibitors tetrabenazine (*n* = 7), valbenazine (*n* = 3), and deutetrabenazine (*n* = 2). Tetrabenazine and valbenazine were mentioned in guidelines from the UK, Germany, and Spain, while deutetrabenazine was mentioned in guidelines from the UK and Germany. Although available in the US and a few other countries, the VMAT2 inhibitors valbenazine and deutetrabenazine [26, 27] are not currently approved for the treatment of TD in the UK or EU (European Medicines Agency). Mentions of GABAergic agonists/benzodiazepines (*n* = 3), amantadine (*n* = 3), and calcium antagonists (*n* = 3) were also identified (Figure 3). Only five mentions of pharmacological (add-on) treatments were recommendations, none of which were ranked. Tetrabenazine (*n* = 3), amantadine (*n* = 1), and buspirone (*n* = 1) were the only three pharmaceutical (add-on) treatments recommended specifically for TD. Tetrabenazine is the only one of these three agents approved for TD in some European countries. Amantadine and buspirone are indicated for Parkinson’s disease and anxiety disorders, respectively.

Fifteen of the 112 TD-related mentions referred to “non-pharmacological or alternative treatments.” None of the mentions of this high-level category were recommendations; all were treatment descriptions. Most (11 of 15) appeared in guideline documents from Germany, and most frequently referred to deep brain stimulation, vitamin E, vitamin B, and ginkgo biloba.

Ten of the 112 TD-related mentions referred to “changing concomitant treatments” (i.e., stopping or decreasing the dose of anticholinergic drugs). Five of these mentions were unranked recommendations (UK, *n* = 4; Spain, *n* = 1), while five were descriptions (UK, *n* = 2; Germany, *n* = 2; Spain, *n* = 1).

### Subgroup analysis of literature informing guideline recommendations on the use of tetrabenazine and tiapride

Publications providing clinical evidence on tetrabenazine and tiapride were identified and are summarized in Table 3. Sixteen studies referenced tetrabenazine, with publication years ranging from 1972 to 2010. The majority (*n* = 13) of these reports were rated as “3” per the SIGN grading system, mostly because they summarized data from nonrandomized trials. Most of these studies had small sample sizes (mean of 35 patients; systematic reviews were not included in the mean calculation), and they did not always exclusively study patients with TD; rather, patients with TD were often a subgroup of a broader study population, such as patients treated with APs or patients with a variety of extrapyramidal symptoms. There was only one publication on tiapride, a randomized, double-blind, controlled, crossover trial of patients with TD (rated as “1−” per the SIGN grading system).

**Table 3.** Summary of studies for tetrabenazine and tiapride

References	Publication year	Study design	Number of patients (TD only, if multiple groups)	Rating^a^
Tetrabenazine				
Asher et al. [28]	1981	Double blind, crossover	10	3
Fahn et al. [29]	1983	Open label, prospective	14	3
Fahn et al. [30]	1985	Case series	6	3
Godwin-Austen et al. [31]	1971	Double blind, control	6	3
Guay [32]	2010	Systematic review	200+ (pooled)	1+
Jankovic [33]	1982	Double blind, crossover	4	1−
Jankovic et al. [34]	1988	Open label, prospective	44	3
Jankovic et al. [35]	1997	Open label	94	3
Kazamatsuri et al. [36]	1972	Crossover trial, evaluator blinded	24	1−
Kazamatsuri et al. [37]	1973	Prospective	13	3
Kenney et al. [38]	2007	Retrospective review	149	3
Kingston [39]	1979	Single-blind case series	6	3
Ondo et al. [40]	1999	Single-blind case series	20	3
Paleacu et al. [41]	2004	Retrospective review	17	3
Stacy et al. [42]	1993	Retrospective review	100	3
Watson et al. [43]	1988	Case series	23	3
Tiapride				
Buruma et al. [44]	1982	Randomized, double-blind, controlled, crossover trial	12	1−

Abbreviation: TD, tardive dyskinesia.

a Scottish Intercollegiate Guidelines Network rating scale was used; a lower rating indicates higher-quality evidence.

## Discussion

The results of this literature review demonstrate that there are no clinical practice guidelines specific to TD management with recommendations in France, Germany, Italy, Spain, or the UK, indicating the need for the development of comprehensive clinical practice recommendations in these countries. Although there were recommendations specific to TD, these were often mentioned in guidelines for schizophrenia, major depressive disorder, or bipolar disorder, and many were not supported by high-quality evidence. The most frequently recommended approach to TD was modification of the AP regimen, though the strength of these recommendations varied. Other recommended or described approaches included pharmacological (add-on) treatments, such as tetrabenazine, GABAergic agonists, amantadine, and calcium antagonists.

Given the high number of AP-treated patients with TD symptoms [7], it is surprising that no TD-specific guidelines with recommendations were identified in EU4 + UK. Clinical practice guidelines can improve outcomes in mental health care across several indicators [45], while the lack of country-specific guidelines may leave healthcare providers without a standardized framework for diagnosing, treating, and managing TD. Most of the existing clinical practice guidelines that mentioned TD focused primarily on schizophrenia. Yet, use of APs has expanded to other health conditions, including bipolar disorder, major depressive disorder, aggression/agitation, and tic disorders (in addition to many off-label uses). Since a considerable number of patients with TD are affected by nonpsychotic disorders [46–48] and because the clinicians (e.g., neurologists) who treat these patients may not consult schizophrenia guidelines, there is a need for TD-specific guidelines. At the very least, management strategies should be included in the guidelines for health conditions for which TD is a risk.

Furthermore, clinical practice guidelines in France, Germany, Spain, and the UK were updated within the last 5 years, while those for Italy have not been updated since 2007. In the US, where new VMAT2 inhibitors have been launched, guidelines and recommendations for treatment have been more recently updated [20, 23] to reflect strong recommendations supported by high-quality evidence for valbenazine and deutetrabenazine. A systematic search of clinical practice guidelines found that these guidelines should be reviewed and updated every 3–5 years to ensure they reflect current evidence [49]. Guidelines in Europe may be subject to updates as new TD treatments become available.

Despite not being part of the EU4 + UK and falling outside the timeframe of this search, an interdisciplinary group from Poland published expert recommendations on the management of drug-induced TD in 2024 [50]. Although not available in the EU, the Polish authors recognized valbenazine and deutetrabenazine as the only agents with class A evidence available; data on other pharmaceutical (add-on) treatments for TD were graded class B or lower. Thus, the findings of the Polish expert group are aligned with those of the present analysis.

The clinical practice guidelines identified in this study do not portray a clear standard of care or treatment strategy for TD. The large variation in treatment options for TD in France, Germany, Italy, Spain, and the UK, coupled with the lack of robust supporting evidence, could pose significant challenges for clinicians and patients. This uncertainty can lead to inconsistent treatment approaches, with some patients potentially receiving suboptimal care. In addition, payer organizations rely on evidence-based medicine for making resource allocation decisions in order to maximize both health benefit and monetary value [51]. The absence of strong clinical data exacerbates the difficulty in determining the most effective interventions, potentially leading to a trial-and-error process that can delay improvement and negatively impact patient quality of life. These conclusions are supported by the findings of a recent systematic literature review and network meta-analysis [52], which showed that most (add-on) TD treatments had low-quality supporting evidence, and that more treatment options and higher-quality trials are needed. These findings further underscore the need for more research validating and informing real-world clinical practice.

### Limitations

When interpreting the results of this review, several limitations need to be considered. First, it is possible that not all EU4 + UK clinical practice guidelines were identified, although the definition of what constituted a “clinical practice guideline” was broad. Second, this broad definition led to the inclusion of documents that are not typical clinical practice guidelines (e.g., German continuing medical education, Spanish peer-reviewed publication); however, the exclusion of these types of documents would not have substantially changed our findings. Third, documents containing recommendations for TD management could have been missed due to our search strategies, which were restricted to at least one EU4 + UK country. Therefore, the *Maudsley Prescribing Guidelines in Psychiatry* and several Cochrane review articles were excluded, which may have restricted the breadth of treatments identified. Additionally, by searching specifically for terms like “TD” and “extrapyramidal symptoms,” we missed indirect references to TD in schizophrenia guidelines released by the French Haute Autorité de Santé, which broadly referred to “intolerance” to APs as justification for a switch in treatment (i.e., no specific reference to the cause of this “intolerance”) [53].

## Implications

Clinical practice guidelines should integrate new evidence and address the quality of supporting evidence when developing treatment recommendations. This goal could be accomplished by explicitly indicating the quality of evidence, providing rankings for recommendations, or prioritizing recommendations with higher quality and more up-to-date evidence behind them. The findings of this review create an opportunity to raise overall awareness of the prevention, screening, diagnosis, treatment, and management of TD. The specific unmet needs identified could drive the development of consensus statements and recommendations that could eventually provide a standardized framework for early detection, timely intervention, and appropriate assessment of treatment. Although two VMAT2 inhibitors (valbenazine and deutetrabenazine) with grade A recommendation are available in the US, Australia, and other countries, they have not yet been approved in the EU and UK. Their approval and availability in the EU and UK would facilitate the treatment of patients with TD with level A evidence pharmacological treatment options and inform the development of TD-specific guidelines in EU4 + UK.

## Conclusions

No TD-specific clinical practice guidelines featuring formal treatment recommendations were identified in EU4 + UK. While TD treatment was mentioned in some guidelines for schizophrenia, major depressive disorder, and bipolar disorder, the majority of the recommendations lacked grading, as they were not supported by high-quality evidence.

## Supporting information

10.1192/j.eurpsy.2025.10047.sm001 Edwards et al. supplementary material Edwards et al. supplementary material

## Data Availability

The datasets used and/or analyzed for the study described in this manuscript are available upon reasonable request. Qualified researchers may request access to study documents, including the study report. Please email USMedInfo@tevapharm.com to make your request.

## Supplementary material

The supplementary material for this article can be found at http://doi.org/10.1192/j.eurpsy.2025.10047.
