# Supplementary material for: Clinical practice guidelines for the treatment of tardive dyskinesia in Europe: A descriptive review
Source: Eur Psychiatry. 2025 Jul 17;68(1):e115. doi: 10.1192/j.eurpsy.2025.10047 (PMC12438997; doi:10.1192/j.eurpsy.2025.10047)
Supplement: Edwards et al. supplementary material [file S0924933825100473sup001.docx]

**Clinical practice guidelines for the treatment of tardive dyskinesia in Europe: a descriptive review**

Mark J. Edwards,^1^ Pierre Michel Llorca,^2–4^ Maurice T. Driessen,^5^ Krzysztof Duma,^6^ Nayla Chaijale,^7^ Liza Sopina,^8^ Sameer Kotak,^9^ Andrea Fagiolini,^10^ David Taylor,^11^ Christoph Correll^12–15^

*^1^Department of Basic and Clinical Neuroscience, Institute of Psychiatry, Psychology and Neuroscience, King’s College London, London, UK; ^2^Fondation FondaMental, Créteil, France; ^3^Clermont-Ferrand University Hospital Center, Clermont-Ferrand, France; ^4^University of Clermont Auvergne, Clermont-Ferrand, France; ^5^Global Health Economics and Outcomes Research, Teva Pharmaceuticals EU, Amsterdam, Netherlands; ^6^Movement Disorders, Teva Pharmaceuticals EU, Amsterdam, Netherlands; ^7^Global Medical Affairs, Teva Branded Pharmaceutical Products R&D, Inc., West Chester, PA, United States; ^8^Independent Consultant, Odense, Denmark; ^9^Yorker Health, Glen Rock, NJ, United States; ^10^Department of Molecular and Developmental Medicine, University of Siena, Siena, Italy; ^11^Pharmaceutical and Public Health Policy, King’s College London, London, UK; ^12^The Zucker Hillside Hospital, Northwell Health, Glen Oaks, NY, United States; ^13^Donald and Barbara Zucker School of Medicine at Hofstra/Northwell, Hempstead, NY, United States; ^14^Feinstein Institutes for Medical Research, Institute of Behavioral Science, Manhasset, NY, United States; ^15^Charité – Universitätsmedizin Berlin, Department of Child and Adolescent Psychiatry, Psychiatry, Berlin, Germany*

# SUPPLEMENTAL MATERIALS

## Supplementary Table 1. Clinical practice guideline sources and documents

| **Country** | **Category** | **Source** | **Title of document or webpage** |
| --- | --- | --- | --- |
| **France** | **Government/Public Agencies for Clinical Guidelines** | Haute Autorité de Santé (HAS) | Protocole National de Diagnostic et de Soins (PNDS) Schizophrénie à début précoce (National diagnostic and care protocol early-onset schizophrenia) |
|  |  | Haute Autorité de Santé (HAS) | ACTES ET PRESTATIONS AFFECTION DE LONGUE DURÉE Schizophrénies (Acts and services long-term condition schizophrenias) |
|  | **Reimbursement Agency** | Haute Autorité de Santé (HAS) | – |
|  | **Medical Association** | French Medical Association (Conseil National de l'Ordre des Médecins) | – |
|  | **Psychiatric Association** | The Fédération Française de Psychiatrie | – |
|  | **Neurological Association** | L’Association Francaise de Psychiatrie Biologique et de Neuropsychopharmacologie (AFPBN) (French Society for Biological Psychiatry and Neuropsychopharmacology) | – |
|  | **Research Organizations/Institutions** | Inserm (Research Institute) | – |
|  | **Patient Organizations/NGOs** | Mental Health Foundation | – |
| **Germany** | **Government/Public Agencies for Clinical Guidelines** | Federal Joint Committee (Gemeinsamer Bundesausschuss or G-BA) | – |
|  | **Reimbursement Agency** | Arzneimittelmarkt-Neuordnungsgesetz (AMNOG) submissions to G-BA; administers AMNOG) | – |
|  |  | German National Institute for Quality and Efficiency in Health Care (IQWiG; Qualität und Wirtschaftlichkeit im Gesundheitswesen) | – |
|  | **Physician Association** | Continuing Medical Education Agency (www.arztcme.de), certified by a state physician association | Spätdyskinesien (Tardive Dyskinesien) Update |
|  |  | Working Group of Scientific Medical Societies (AWMF) | Dystonie – Leitlinien für Diagnostik und Therapie in der Neurologie (Dystonia - guidelines for diagnostics and therapy in neurology) |
|  |  | German Medical Association (Bundesärztekammer) | – |
|  | **Psychiatric Association** | German Society for Psychiatry, Psychotherapy and Neurology (DGPPN) | S3 Guideline for Schizophrenia |
|  |  |  | S3-Leitlinie zur Diagnostik und Therapie Bipolarer Störungen (S3 guidelines for diagnosis and treatment of bipolar disorder) |
|  | **Other Professional Organization** | German Society for Biological Psychiatry (DGBP) | – |
|  |  | German Medical Journal Deutsches Ärzteblatt International | Hasan A, Falkai P, Lehmann I, Gaebel W: Clinical practice guideline: Schizophrenia. Dtsch Arztebl Int 2020; 117: 412–9. DOI: 10.3238/arztebl.2020.0412 |
|  |  | Community Psychiatry Association | – |
|  | **Patient Organizations/NGOs** | Mental Health Medical Association | – |
| **Italy** | **Government/Public Agencies for Clinical Guidelines** | National Institute of Health (Istituto Superiore di Sanità or ISS) | The Italian guidelines for early intervention in schizophrenia: development and conclusions |
|  |  | National guidelines depository (Sistema Nazionale Linee Guida, SNLG) | – |
|  | **Reimbursement Agency** | Italian Medicines Agency (AIFA) | – |
|  | **Medical Association** | Italian Society of Internal Medicine | – |
|  | **Psychiatric Association** | Italian Society of Psychiatry (Societa Italiana di Psichiatria, SIP) | – |
|  |  | Italian Society of Biological Psychiatry (Societa Italian di Psichiatria Biologica, SIBP) | – |
|  | **Neurological Association** | Associazione Italiana Neuropatologia e Neurobiologia Clinica | – |
|  | **Other Professional Organisation** | Italian Society of Psychopathology (& their journal) | – |
| **Spain** | **Government/Public Agencies for Clinical Guidelines** | Spanish National Health System's Clinical Practice Guidelines Repository | GUÍA CLÍNICA PARA EL TRATAMIENTO DE LA ESQUIZOFRENIA (Clinical guidelines for treatment of schizophrenia) |
|  |  |  | Clinical practice gideline for schizophrenia and incipient psychotic disorder |
|  | **Reimbursement Agency** | Spanish Ministry of Health | – |
|  |  | The Spanish Medicines Agency of Medicinal Products and Medical Devices (ie, Agencia Española de Medicamentos y Productos Sanitarios; AEMPS) | – |
|  | **Medical Association** | Medical Association in Spain | – |
|  |  | Medical Journal - Psiquiatría Biológica (Biological Psychiatry) | Tardive dyskinesia. A clinical and therapeutic review |
|  | **Psychiatric Association** | Spanish Society of Psychiatry (Sociedad Española de Psiquiatría; SEPSM) | Tratamiento Farmacológico y Psicológico de los Pacientes adultos con un Trastorno Mental Grave y un Trastorno por Uso de Sustancias (Pharmacological and psychological treatment of adult patients with a severe mental disorder and a substance use disorder) |
|  |  | Spanish National Health System's Clinical Practice Guidelines Repository | Guía de Práctica Clínica sobre Trastorno Bipolar (Clinical practice guideline on bipolar disorder) |
|  |  | Spanish Society of Psychiatry and the Spanish Society of Biological Psychiatry | Recommendations for switching antipsychotics. A position statement of the Spanish Society of Psychiatry and the Spanish Society of Biological Psychiatry |
|  | **Neurological Association** | Spanish Society of Neurology (Sociedad Española de Neurología; SEN) | – |
|  | **Other Professional Organization** | SEFH (Sociedas Espanola de Farmacia Hospitalaria; Spanish Society of Hospital Pharmacy) | GUÍA DE UTILIZACIÓN DE ANTIPSICÓTICOS (Guide for use of antipsychotics) |
|  |  | The Spanish Association of Neuropsychiatry (AEN) | – |
|  | **Patient Organizations/NGOs** | Spanish Mental Health Confederation | – |
| **UK** | **Government/Public Agencies for Clinical Guidelines** | National Health Service (NHS) | Antipsychotics – prescribing guideline (prescribing guideline for primary care prescribers) |
|  |  | Scottish Intercollegiate Guidelines Network (SIGN) | Management of schizophrenia: SIGN publication 131. 2013 |
|  |  | National Health Service (NHS) Essex | Section 9: Management of movement disorders and extrapyramidal side effects |
|  |  | National Health Service (NHS) Glasgow | Extrapyramidal side effects - management |
|  |  | National Health Service (NHS) Hertfordshire | Guidelines on choice and selection of antipsychotics for the management of psychosis and schizophrenia in adults |
|  |  | National Health Service (NHS) Kent | Online formulary/antipsychotics |
|  |  | National Health Service (NHS) Leeds | Medicines Management & Pharmacy Services (MMPS); Extrapyramidal side-effects of antipsychotic drug treatment |
|  |  | National Health Service (NHS) Scotland | Extrapyramidal side effects- Management |
|  | **Reimbursement Agency** | National Institute for Health and Care Excellence (NICE) | Psychosis and schizophrenia in adults: prevention and management |
|  |  | National Institute for Health and Care Excellence (NICE) | Psychosis and schizophrenia in children and young people: recognition and management |
|  |  | National Institute for Health and Care Excellence (NICE) | Bipolar disorder: The NICE giudelin on the assessment and management of bipolar disorder in adults, children and young people in primary and secondary care |
|  |  | National Institute for Health and Care Excellence (NICE) | Bipolar disorder: Antipsychotics [prescribing information] |
|  |  | National Institute for Health and Care Excellence (NICE) | Psychosis and schizophrenia: Adverse effects [prescribing information] |
|  |  | Scottish Medicines Consortium | – |
|  | **Medical Association** | British Medical Association (BMA) | – |
|  | **Psychiatric Association** | The British Association for Psychopharmacology (BAP) | Evidence-based guidelines for the pharmacological treatment of schizophrenia: updated recommendations from the British Association for Psychopharmacology^a^ |
|  | **Neurological Association** | British Neurological Society (BNS) | – |
|  | **Other Professional Organisation** | Royal College of Psychiatrists | – |
|  | **Patient Organisations/NGOs** | Living with Schizophrenia UK | – |
|  |  | Mental Health UK | – |
|  |  | Mind | – |

^a^This guideline focuses on schizophrenia and bipolar disorder, and is counted twice (ie, two different guidelines).

## Supplementary Table 2. Search strategy for clinical practice guidelines on MEDLINE (via PubMed) January 2000 – February 2025

| Search | Query |
| --- | --- |
| 1 | (UK OR "United Kingdom" OR England OR Britain OR "Great Britain" OR Wales OR Scotland OR "Northern Ireland") AND (Tardive Dyskinesia[Title/Abstract]) OR (("Tardive Dyskinesia"[53]) OR (("Tardive Dyskinesia/chemically induced"[53] OR "Tardive Dyskinesia/drug therapy"[53] OR "Tardive Dyskinesia/etiology"[53] OR "Tardive Dyskinesia/prevention and control"[53] OR "Tardive Dyskinesia/therapy"[53] ))) AND ((booksdocs[Filter] OR meta-analysis[Filter] OR review[Filter] OR systematicreview[Filter]) AND (2000:2023[pdat]))  (UK OR "United Kingdom" OR England OR Britain OR "Great Britain" OR Wales OR Scotland OR "Northern Ireland") AND (Tardive Dyskinesia[Title/Abstract]) OR (("Tardive Dyskinesia"[Mesh]) OR (("Tardive Dyskinesia/chemically induced"[Mesh] OR "Tardive Dyskinesia/drug therapy"[Mesh] OR "Tardive Dyskinesia/etiology"[Mesh] OR "Tardive Dyskinesia/prevention and control"[Mesh] OR "Tardive Dyskinesia/therapy"[Mesh] ))) AND (2023:2025[pdat])) |
| 2 | (Spain OR Spanish) AND (Tardive Dyskinesia[Title/Abstract]) OR (("Tardive Dyskinesia"[53]) OR (("Tardive Dyskinesia/chemically induced"[53] OR "Tardive Dyskinesia/drug therapy"[53] OR "Tardive Dyskinesia/etiology"[53] OR "Tardive Dyskinesia/prevention and control"[53] OR "Tardive Dyskinesia/therapy"[53] ))) AND ((booksdocs[Filter] OR meta-analysis[Filter] OR review[Filter] OR systematicreview[Filter]) AND (2000:2023[pdat])) AND (booksdocs[Filter] OR meta-analysis[Filter] OR review[Filter] OR systematicreview[Filter])  (Spain OR Spanish) AND (Tardive Dyskinesia[Title/Abstract]) OR (("Tardive Dyskinesia"[Mesh]) OR (("Tardive Dyskinesia/chemically induced"[Mesh] OR "Tardive Dyskinesia/drug therapy"[Mesh] OR "Tardive Dyskinesia/etiology"[Mesh] OR "Tardive Dyskinesia/prevention and control"[Mesh] OR "Tardive Dyskinesia/therapy"[Mesh] ))) AND (2023:2025[pdat])) |
| 3 | (Italy OR Italian) AND (Tardive Dyskinesia[Title/Abstract]) OR (("Tardive Dyskinesia"[53]) OR (("Tardive Dyskinesia/chemically induced"[53] OR "Tardive Dyskinesia/drug therapy"[53] OR "Tardive Dyskinesia/etiology"[53] OR "Tardive Dyskinesia/prevention and control"[53] OR "Tardive Dyskinesia/therapy"[53] ))) AND ((booksdocs[Filter] OR meta-analysis[Filter] OR review[Filter] OR systematicreview[Filter]) AND (2000:2023[pdat])) AND (booksdocs[Filter] OR meta-analysis[Filter] OR review[Filter] OR systematicreview[Filter]) AND (booksdocs[Filter] OR meta-analysis[Filter] OR review[Filter] OR systematicreview[Filter])  (Italy OR Italian) AND (Tardive Dyskinesia[Title/Abstract]) OR (("Tardive Dyskinesia"[Mesh]) OR (("Tardive Dyskinesia/chemically induced"[Mesh] OR "Tardive Dyskinesia/drug therapy"[Mesh] OR "Tardive Dyskinesia/etiology"[Mesh] OR "Tardive Dyskinesia/prevention and control"[Mesh] OR "Tardive Dyskinesia/therapy"[Mesh] ))) AND (2023:2025[pdat])) |
| 4 | (France OR French) AND (Tardive Dyskinesia[Title/Abstract]) OR (("Tardive Dyskinesia"[53]) OR (("Tardive Dyskinesia/chemically induced"[53] OR "Tardive Dyskinesia/drug therapy"[53] OR "Tardive Dyskinesia/etiology"[53] OR "Tardive Dyskinesia/prevention and control"[53] OR "Tardive Dyskinesia/therapy"[53] ))) AND ((booksdocs[Filter] OR meta-analysis[Filter] OR review[Filter] OR systematicreview[Filter]) AND (2000:2023[pdat])) AND (booksdocs[Filter] OR meta-analysis[Filter] OR review[Filter] OR systematicreview[Filter]) AND (booksdocs[Filter] OR meta-analysis[Filter] OR review[Filter] OR systematicreview[Filter]) AND (booksdocs[Filter] OR meta-analysis[Filter] OR review[Filter] OR systematicreview[Filter])  (France OR French) AND (Tardive Dyskinesia[Title/Abstract]) OR (("Tardive Dyskinesia"[Mesh]) OR (("Tardive Dyskinesia/chemically induced"[Mesh] OR "Tardive Dyskinesia/drug therapy"[Mesh] OR "Tardive Dyskinesia/etiology"[Mesh] OR "Tardive Dyskinesia/prevention and control"[Mesh] OR "Tardive Dyskinesia/therapy"[Mesh] ))) AND (2023:2025[pdat])) |
| 5 | (Germany OR German) AND (Tardive Dyskinesia[Title/Abstract]) OR (("Tardive Dyskinesia"[53]) OR (("Tardive Dyskinesia/chemically induced"[53] OR "Tardive Dyskinesia/drug therapy"[53] OR "Tardive Dyskinesia/etiology"[53] OR "Tardive Dyskinesia/prevention and control"[53] OR "Tardive Dyskinesia/therapy"[53] ))) AND ((booksdocs[Filter] OR meta-analysis[Filter] OR review[Filter] OR systematicreview[Filter]) AND (2000:2023[pdat])) AND (booksdocs[Filter] OR meta-analysis[Filter] OR review[Filter] OR systematicreview[Filter]) AND (booksdocs[Filter] OR meta-analysis[Filter] OR review[Filter] OR systematicreview[Filter]) AND (booksdocs[Filter] OR meta-analysis[Filter] OR review[Filter] OR systematicreview[Filter]) AND (booksdocs[Filter] OR meta-analysis[Filter] OR review[Filter] OR systematicreview[Filter])  (Germany OR German) AND (Tardive Dyskinesia[Title/Abstract]) OR (("Tardive Dyskinesia"[Mesh]) OR (("Tardive Dyskinesia/chemically induced"[Mesh] OR "Tardive Dyskinesia/drug therapy"[Mesh] OR "Tardive Dyskinesia/etiology"[Mesh] OR "Tardive Dyskinesia/prevention and control"[Mesh] OR "Tardive Dyskinesia/therapy"[Mesh] ))) AND (2023:2025[pdat])) |
| 6 | (((((Germany OR German) AND (Tardive Dyskinesia[Title/Abstract]) OR (("Tardive Dyskinesia"[53]) OR (("Tardive Dyskinesia/chemically induced"[53] OR "Tardive Dyskinesia/drug therapy"[53] OR "Tardive Dyskinesia/etiology"[53] OR "Tardive Dyskinesia/prevention and control"[53] OR "Tardive Dyskinesia/therapy"[53] ))) AND ((booksdocs[Filter] OR meta-analysis[Filter] OR review[Filter] OR systematicreview[Filter]) AND (2000:2023[pdat])) AND (booksdocs[Filter] OR meta-analysis[Filter] OR review[Filter] OR systematicreview[Filter]) AND (booksdocs[Filter] OR meta-analysis[Filter] OR review[Filter] OR systematicreview[Filter]) AND (booksdocs[Filter] OR meta-analysis[Filter] OR review[Filter] OR systematicreview[Filter]) AND (booksdocs[Filter] OR meta-analysis[Filter] OR review[Filter] OR systematicreview[Filter]) AND (booksdocs[Filter] OR meta-analysis[Filter] OR review[Filter] OR systematicreview[Filter])) OR ((France OR French) AND (Tardive Dyskinesia[Title/Abstract]) OR (("Tardive Dyskinesia"[53]) OR (("Tardive Dyskinesia/chemically induced"[53] OR "Tardive Dyskinesia/drug therapy"[53] OR "Tardive Dyskinesia/etiology"[53] OR "Tardive Dyskinesia/prevention and control"[53] OR "Tardive Dyskinesia/therapy"[53] ))) AND ((booksdocs[Filter] OR meta-analysis[Filter] OR review[Filter] OR systematicreview[Filter]) AND (2000:2023[pdat])) AND (booksdocs[Filter] OR meta-analysis[Filter] OR review[Filter] OR systematicreview[Filter]) AND (booksdocs[Filter] OR meta-analysis[Filter] OR review[Filter] OR systematicreview[Filter]) AND (booksdocs[Filter] OR meta-analysis[Filter] OR review[Filter] OR systematicreview[Filter]) AND (booksdocs[Filter] OR meta-analysis[Filter] OR review[Filter] OR systematicreview[Filter]))) OR ((Italy OR Italian) AND (Tardive Dyskinesia[Title/Abstract]) OR (("Tardive Dyskinesia"[53]) OR (("Tardive Dyskinesia/chemically induced"[53] OR "Tardive Dyskinesia/drug therapy"[53] OR "Tardive Dyskinesia/etiology"[53] OR "Tardive Dyskinesia/prevention and control"[53] OR "Tardive Dyskinesia/therapy"[53] ))) AND ((booksdocs[Filter] OR meta-analysis[Filter] OR review[Filter] OR systematicreview[Filter]) AND (2000:2023[pdat])) AND (booksdocs[Filter] OR meta-analysis[Filter] OR review[Filter] OR systematicreview[Filter]) AND (booksdocs[Filter] OR meta-analysis[Filter] OR review[Filter] OR systematicreview[Filter]) AND (booksdocs[Filter] OR meta-analysis[Filter] OR review[Filter] OR systematicreview[Filter]))) OR ((Spain OR Spanish) AND (Tardive Dyskinesia[Title/Abstract]) OR (("Tardive Dyskinesia"[53]) OR (("Tardive Dyskinesia/chemically induced"[53] OR "Tardive Dyskinesia/drug therapy"[53] OR "Tardive Dyskinesia/etiology"[53] OR "Tardive Dyskinesia/prevention and control"[53] OR "Tardive Dyskinesia/therapy"[53] ))) AND ((booksdocs[Filter] OR meta-analysis[Filter] OR review[Filter] OR systematicreview[Filter]) AND (2000:2023[pdat])) AND (booksdocs[Filter] OR meta-analysis[Filter] OR review[Filter] OR systematicreview[Filter]) AND (booksdocs[Filter] OR meta-analysis[Filter] OR review[Filter] OR systematicreview[Filter]))) OR ((UK OR "United Kingdom" OR England OR Britain OR "Great Britain" OR Wales OR Scotland OR "Northern Ireland") AND (Tardive Dyskinesia[Title/Abstract]) OR (("Tardive Dyskinesia"[53]) OR (("Tardive Dyskinesia/chemically induced"[53] OR "Tardive Dyskinesia/drug therapy"[53] OR "Tardive Dyskinesia/etiology"[53] OR "Tardive Dyskinesia/prevention and control"[53] OR "Tardive Dyskinesia/therapy"[53] ))) AND ((booksdocs[Filter] OR meta-analysis[Filter] OR review[Filter] OR systematicreview[Filter]) AND (2000:2023[pdat])) AND (booksdocs[Filter] OR meta-analysis[Filter] OR review[Filter] OR systematicreview[Filter])) |

## Supplementary Table 3. Levels of evidence of individual sources according to SIGN [24]

| **Level** | **Operationalisation** |
| --- | --- |
| 1++ | High-quality meta-analyses, systematic reviews of RCTs, or RCTs with a very low risk of bias |
| 1+ | Well-conducted meta-analyses, systematic reviews, or RCTs with a low risk of bias |
| 1- | High risk of bias meta-analyses/RCTs |
| 2++ | High-quality systematic reviews of case control or cohort studies;  High-quality case control or cohort studies with a very low risk of confounding or bias and a high probability that the relationship is causal |
| 2+ | Well-conducted case control or cohort studies with a low risk of confounding or bias and a moderate probability that the relationship is causal |
| 2- | Case control or cohort studies with a high risk of confounding or bias and a significant risk that the relationship is not causal |
| 3 | Non-analytic studies (eg case reports, case series) |
| 4 | Expert opinion |

Abbreviations: RCT, randomised controlled trial; SIGN, Scottish Intercollegiate Guidelines Network.

## Supplementary Table 4. Treatment recommendations grading system according to SIGN [24]

| **Grade** | **Operationalisation** |
| --- | --- |
| **A** | At least one meta-analysis, systematic review, or randomised clinical trial rated as 1++, and directly applicable to the target population; or a body of evidence consisting principally of studies rated as 1+, directly applicable to the target population, and demonstrating overall consistency of results |
| **B** | A body of evidence including studies rated as 2++, directly applicable to the target population, and demonstrating overall consistency of results; or extrapolated evidence from studies rated as 1++ or 1+ |
| **C** | A body of evidence including studies rated as 2+, directly applicable to the target population and demonstrating overall consistency of results; or extrapolated evidence from studies rated as 2++ |
| **D** | Evidence level 3 or 4; or extrapolated evidence from studies rated as 2+ or expert opinion or consensus |
| **NO RANK^a^** | No grade provided for recommendation (an additional grading) |

Abbreviations: SIGN, Scottish Intercollegiate Guidelines Network.

^a^Recommendations were assigned a ‘NO RANK’ rating if they did not meet any of the grading categories in SIGN.

## Supplementary Table 5. List of treatment categories identified in guidelines

| **Treatment category** | **Treatment/strategy** |
| --- | --- |
| **Changing concomitant treatments (non–TD-specific)** | Change/stop antimuscarinic |
|  | Stop/decrease anticholinergic |
| **Modification of antipsychotic regimen (dose change, treatment switch, or cessation)** | Change to injectable |
|  | Discontinue antipsychotic |
|  | Increase antipsychotic dose |
|  | Reduce antipsychotic dose |
|  | Unspecified change/alternative second generation |
|  | Unspecified dose change |
|  | Aripiprazole |
|  | Clozapine |
|  | Olanzapine |
|  | Quetiapine |
|  | Risperidone |
| **Pharmacological (add-on) treatments (ie, targeted at TD symptoms)** | Amantadine |
|  | Buspirone |
|  | Calcium antagonists |
|  | catecholaminergic drugs |
|  | Cholinesterase inhibitors |
|  | Cholinomimetic drugs (cholinergic agonists) |
|  | Deutetrabenazine |
|  | Donepezil/cholinergics |
|  | GABAergic agonists/Benzodiazepines |
|  | Levo-Dopa |
|  | Non-benzodiazepine GABAergic agonists |
|  | Propranolol |
|  | Tetrabenazine |
|  | Tiapride |
|  | Valbenazine |
|  | VMAT2 inhibitor (non-specific) |
| **Other interventions** | Botulinum toxin/Botox |
|  | Branched-chain amino acids |
|  | Clonidine |
|  | Deep brain stimulation |
|  | Electroconvulsive therapy |
|  | Ginko biloba |
|  | Hypnosis/relaxation |
|  | Vitamin B |
|  | Vitamin E |

Abbreviation: TD, tardive dyskinesia.
